# Associations between feeding practices and growth and neurodevelopmental outcomes at 36 months among children living in low- and low-middle income countries who participated in the BRAIN-HIT trial

**DOI:** 10.1186/s40795-018-0228-9

**Published:** 2018-04-25

**Authors:** Barbara T. Do, Nellie I. Hansen, Carla Bann, Rebecca L. Lander, Shivaprasad S. Goudar, Omrana Pasha, Elwyn Chomba, Sangappa M. Dhaded, Vanessa R. Thorsten, Jan L. Wallander, Fred J. Biasini, Richard Derman, Robert L. Goldenberg, Waldemar A. Carlo

**Affiliations:** 1 RTI International, Research Triangle Park, North Carolina USA; 2 University of Colorado School of Medicine, Aurora, Colorado USA; 3 KLE Jawaharlal Nehru Medical College, Belgaum, Karnataka India; 4 Department of Population, Family & Reproductive Health, John Hopkins University Bloomberg School of Public Health, Baltimore, Maryland USA; 5 University of Zambia, Lusaka, Zambia; 6 University of California, Merced, Merced, California USA; 7 Department of Pediatrics/Division of Neonatology, University of Alabama at Birmingham, Birmingham, Alabama USA; 8 Thomas Jefferson University, Philadelphia, Pennsylvania USA; 9 Columbia University, New York, New York USA

**Keywords:** Bayley scores of infant development, Developmental outcome, Stunting, Wasting, Dietary diversity, Low- and middle-income countries

## Abstract

**Background:**

Feeding practices over the first several years of a child’s life can critically influence development. The purpose of this study was to examine associations between feeding practices and growth and neurodevelopmental outcomes at 36 months of age among children from low- and low-middle-income countries (LMIC).

**Methods:**

We conducted a secondary analysis using data collected from children in India, Pakistan, and Zambia who were enrolled in a randomized controlled trial of a home-based early development intervention program called Brain Research to Ameliorate Impaired Neurodevelopment Home-based Intervention Trial. Qualitative dietary data collected at 36 months was used to assess the modified Minimum Acceptable Diet (mMAD), a measure based on a core indicator developed by the World Health Organization to measure whether young children receive the minimum number of meals recommended and adequate diversity of major food groups in their diet. Regression models were used to assess cross-sectional associations between diet and growth indices, including Z-scores for height-for-age (HAZ), weight-for-age (WAZ), weight-for-height (WHZ), head circumference (HCZ), and Bayley Scales of Infant Development II mental and psychomotor developmental measures at 36 months of age.

**Results:**

Of 371 children, 174 (47%) consumed the mMAD, with significantly higher mean adjusted WHZ than those who did not meet mMAD (0.20 vs − 0.08, *p* = 0.05). Egg consumption was found to be significantly associated with a decreased risk of wasting [adjusted RR (95% CI): 0.37 (0.15, 0.89), *p* = 0.03]. HCZ at 36 months did not differ significantly for children who did and did not receive the mMAD.

**Conclusion:**

Meeting the mMAD was associated with better weight-for-height outcomes at 36 months in children in these three LMIC, highlighting the importance of adequate food quantity and quality.

**Trial registration:**

NCT00639184 registered on March 20, 2008.

## Background

According to the World Health Organization (WHO), approximately 45% of the 5.9 million deaths of children under the age of 5 years in 2015 were linked to undernutrition [1]. While undernutrition may not be the main cause of death for children in this age group, it is an important underlying contributing factor that increases a child’s susceptibility to severe diseases and can have a large impact on a surviving child’s developmental outcome. Several studies have shown that poor young child feeding (YCF) practices are a risk factor for wasting, defined as weight-for-height Z-scores < -2SD, often the result of insufficient amounts of food and/or infectious diseases [2–4]. Additionally, as many as one in three children under 5 years in low- and low-middle-income countries (LMIC) are stunted (height-for-age Z-scores < -2SD), due to chronic undernutrition [5]. Stunting has not only been associated with increased risk of mortality, but also heightened risk of morbidity, delayed motor development and impaired cognition, potentially affecting long-term health outcomes [6–8]. Thus, considering the vital role of nutrition in child development, appropriate YCF practices are critical in the physical growth and cognitive development of children living in disadvantaged environments [9].

In 2008, WHO published a set of eight core indicators aimed at improving YCF practices in children 0–23 months of age and, ultimately, child survival [10]. These indicators focus on breastfeeding practices up to 1 year of age and the introduction of complementary foods around 6–8 months, as well as meal frequency and dietary diversity (general indicators of adequate energy and micronutrient intakes, respectively) in children aged 6–23 months.

We used data collected during the Brain Research to Ameliorate Impaired Neurodevelopment Home-based Intervention Trial (BRAIN-HIT) to conduct a secondary analysis to assess associations between YCF practices and growth and developmental outcomes at 36 months of age in children from three LMIC. While the YCF indicators were designed for younger children, this study had the unique opportunity to not only examine potential growth outcomes in slightly older children, but also to explore the relationship between YCF indicators and Bayley Scales of Infant Development – Second Edition (BSID-II) Mental Developmental Index (MDI) and Psychomotor Development Index (PDI) scores. We hypothesized that meeting YCF indicators will have positive associations with growth, and mental and psychomotor developmental outcomes.

## Methods

### Study population

Children studied were enrolled in the FIRST BREATH trial [11] and participated in the follow-on BRAIN-HIT (ClinicalTrials.gov: NCT00639184). BRAIN-HIT was a parallel-randomized controlled trial conducted in rural communities in India, Pakistan, and Zambia from 2007 to 2010 that aimed to determine whether a home-based, parent-provided early developmental intervention (EDI) plus WHO Enhanced Health Education Counseling (HC) would improve the BSID-II MDI scores at 36 months when compared to HC only in infants who have had birth asphyxia. The trial constituted two treatment populations: (1) babies who had mild-moderate birth asphyxia and were resuscitated via bag and mask ventilation and (2) non-resuscitated babies without perinatal complications who served as the healthy comparison group. Mild-moderate birth asphyxia was defined as insufficient breathing at birth and needing positive pressure ventilation. Infants were eligible for BRAIN-HIT if they met the following criteria: (1) weighed at least 1500 g at birth, (2) had a normal neurological examination (Stage I or II on the Ellis scale), and (3) were willing to participate in an intervention program for 36 months. Infants were ineligible if the mother was not contacted within 7 days of giving birth, younger than 15 years of age, unable/unwilling to participate, or not planning to stay in the study communities for the subsequent three years.

BRAIN implemented a modified version of the WHO Integrated Management of Childhood Illnesses Program (IMCI) [12] for the Health and Safety Counseling curriculum used in both arms of the trial. At enrollment and the 2-week visit, demographics and family resources were noted. At 12-, 24- and 36-months of age, information on family resources, health status and growth measurements were collected as well as neurodevelopmental assessments for BSID-II MDI and PDI scores. Child diet information was collected at 36 months of age. Full details of the BRAIN-HIT protocol have been published [13].

**Fig. 1:**
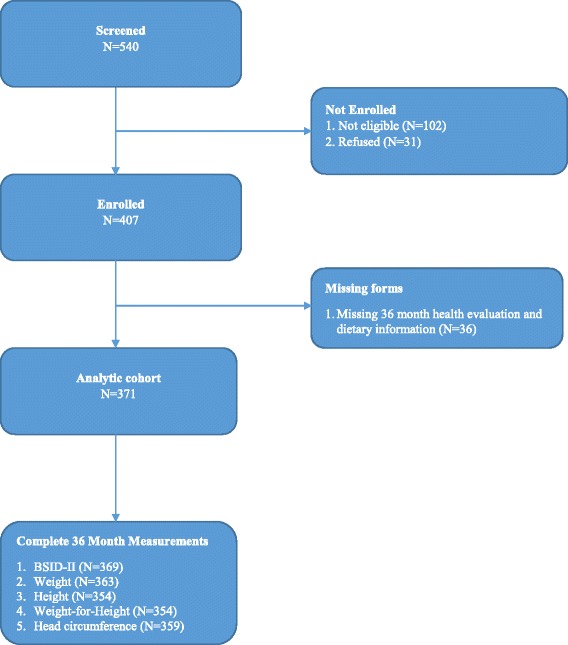
Cohort

Among the 540 infants screened, 438 (81%) were eligible for participation with 407 (93%) of the eligible infants having mothers who consented to participate in the study [14]. Among infants whose mothers consented, 371 (91%) had completed dietary forms and health evaluations at 36 months and are included in the current analysis (Fig. 1).

### Dietary methods and indicators

Young child feeding practices were evaluated during the last visit at 36 months of age by use of a qualitative dietary questionnaire, including number of meals and food groups consumed on an average day. In 2008, WHO published a set of reliable and valid core indicators to assess feeding practices in children up to two years old. Since BRAIN-HIT collected data on dietary practices at 36 months of age only, we applied the WHO recommended diet up to 24 months to the 36 month intake assessment. The three core indicators of interest were modified versions of the Minimum Dietary Diversity (mMDD), Minimum Meal Frequency (mMMF), and Minimum Acceptable Diet (mMAD). Children met the mMDD if they received at least four of the following food groups: (1) grains, roots or tubers; (2) legumes or nuts; (3) dairy products; (4) flesh foods (meat, poultry, or insects); (5) eggs; and (6) fruits or vegetables. BRAIN-HIT did not distinguish between vitamin-A rich and non-vitamin-A rich fruits and vegetables and thus, all fruit and vegetables were combined in one food group. However, the mMDD was considered met if at least four food groups were being regularly consumed in a day, as per the WHO definition. To achieve mMMF, BRAIN-HIT applied the WHO Integrated Management of Childhood Illness (IMCI) program recommendation of three meals with two additional snacks a day for this age group. Finally, children met the mMAD if they met both the mMDD and mMMF.

### Demographics

Data were collected on demographic characteristics of the child (sex, premature birth, birth weight, resuscitation status, exclusively breast fed the first 6 months) and mother (socioeconomic status, age, educational level, marital status, parity).

### Outcomes

#### Anthropometric measures

Three anthropometric measures were taken at each visit: length/height, weight, and head circumference. Weight was measured using digital infant weighing scales in Pakistan and analog scales in India and Zambia. Head circumference was measured using a plasticized or fiberglass non-elastic tape measure. Standing height was measured in Zambia while recumbent length was measured in India and Pakistan. In India, children were made to lie supine on clean level floors with their heads resting against the wall and looking straight at the ceiling. Their legs were straightened at the knee with their feet perpendicular to the leg while ensuring their heads, backs, buttocks, and heels were in contact with the floor. Hard boards were then placed against the foot and lengths were measured by placing the measuring tape from the wall to inner edge of the board. Children were properly restrained during the procedure. In Pakistan, recumbent length was measured two times with a Seca 416 infantometer (Perspective Enterprises, Portage, MI). If the two initial measurements differed by more than 0.2 cm, a third measurement was undertaken. Anthropometric instruments were calibrated regularly.

Weight, length/height, and head circumference measurements taken at 36 months of age were used to determine weight-for-age (WAZ), height-for-age (HAZ), weight-for-height (WHZ) and head circumference-for-age Z-scores (HCZ) based on international growth standards developed by WHO for children up to 5 years of age [15]. Lengths were converted to heights by subtracting 0.7 cm as recommended by WHO [16]. Stunting and wasting were defined as HAZ < -2SD and WHZ < -2SD, respectively.

#### Developmental measures

The BSID-II is a well-validated measure of development in infants aged 1–42 months [17]. Both the BSID-II MDI- and PDI- scores, which measure cognitive development and motor skills, respectively, were used to assess development at 36 months. The BSID-II was used as a main measure for the BRAIN-HIT trial due to its extensive use in a number of LMIC. To verify the validity of BSID-II in the local context, it was pretested at each site and a few items were modified to ensure cultural appropriateness (e.g., image of a sandal in place of a shoe). The BSID-II was administered to each child in the appropriate language using standard material by certified neurodevelopmental evaluators (pediatricians and psychologists familiar with the local language and culture) who were masked to the birth history and intervention group. All evaluators received an intensive 4-day training in the purpose and correct administration of each item.

### Statistical analysis

Descriptive statistics were calculated for child and maternal characteristics. Frequencies and percentages were reported for categorical variables with differences in characteristics between sites tested for by chi-square and Fisher’s exact test. Means, standard deviations, medians, minimums and maximums were reported for continuous variables with difference in means tested using the Kruskal Wallis test.

Linear regression models fitting each BSID-II Score index (i.e. MDI and PDI) and anthropometric measures (i.e. WAZ, HAZ, WHZ, and HCZ) were used to estimate adjusted mean scores in groups of children defined by dietary consumption. Models included one dietary indicator at a time as the primary independent variable with site, intervention group, resuscitation status, socioeconomic status, child sex, exclusively breastfed first 6 months, birth weight, preterm status, maternal age, and maternal education level as covariates. Results from the main trial suggested some heterogeneity between neurodevelopmental evaluator scoring. To account for potential confounding due to evaluator, evaluator within site was included as a nested effect in the BSID-II models. Statistical significance for a difference in adjusted means for those with and without each dietary indicator was determined by the F test. Tests of interaction were conducted to assess whether the relationship between the dietary indicator and outcome differed between the three study sites with means shown by site if interactions were significant.

Adjusted relative risks (RR) and 95% confidence intervals (CI) for stunting and wasting were estimated using Poisson regression models with robust variance estimators [18] assuming an independent correlation structure. Relative risks were adjusted for site, intervention group, resuscitation status, and child and maternal characteristics with statistical significance determined by Wald chi-square tests. All analyses were conducted in SAS version 9.4.

## Results

Maternal average age among study participants was 24.9 years but differed significantly among each country (India, 22.8 years; Zambia, 25.2 years; Pakistan, 27.4 years; *p* < 0.01) (Table 1). The majority (97%) of mothers from Pakistan received no formal education, compared to 38% from India and 7% from Zambia (*p* < 0.01). A larger proportion of women from Pakistan had preterm babies (29%) than from India and Zambia (8% and 3%, *p* < 0.01). Infants from Zambia had heavier average birth weights (3159 g) compared to India (2694 g) and Pakistan (2517 g) (*p* < 0.01). Notably, fewer children from Pakistan were exclusively breastfed in the first 6 months (10%) compared to India (83%) and Zambia (96%) (*p* < 0.01).

**Table 1:** Study Participant and Maternal Characteristics, Food Group Consumption and Young Child Feeding Index Components by Site

Measures	Subgroup	Statistic	All study participants *N* = 371	Zambia *N* = 92	India *N* = 159	Pakistan *N* = 120	*P*-value*
Study participant characteristics							
Intervention group	Early developmental intervention	*n* (%)	186 (50)	44 (48)	80 (50)	62 (52)	0.86
	Control		185 (50)	48 (52)	79 (50)	58 (48)	
Child sex	Male	*n* (%)	218 (60)	53 (58)	96 (60)	69 (61)	0.87
	Female		146 (40)	39 (42)	63 (40)	44 (39)	
Premature birth	Yes	*n* (%)	50 (14)	3 (3)	12 (8)	35 (29)	< 0.01
	No		318 (86)	87 (97)	146 (92)	85 (71)	
Birth weight(g)		N	360	92	159	109	
		Mean (SD)	2760 (504)	3159 (444)	2694 (367)	2517 (528)	< 0.01
		Median	2700	3100	2600	2500	
		Min, Max	1500, 4500	2000, 4500	1600, 3800	1500, 3600	
Resuscitated	Yes	*n* (%)	154 (42)	35 (38)	80 (50)	39 (33)	< 0.01
	No		217 (58)	57 (62)	79 (50)	81 (68)	
Exclusively breastfed first 6 months	Yes	*n* (%)	232 (63)	88 (96)	132 (83)	12 (10)	< 0.01
	No		139 (37)	4 (4)	27 (17)	108 (90)	
Maternal characteristics							
SES by wealth index terciles	Low (SES score < = − 1.3)	*n* (%)	176 (47)	28 (30)	74 (47)	74 (62)	< 0.01
	Medium (−1.3 < SES score < 2.8)		162 (44)	42 (46)	77 (48)	43 (36)	
	High (SES score > =2.8)		33 (9)	22 (24)	8 (5)	3 (3)	
Maternal age (years)		*N*	371	92	159	120	
		Mean (SD)	24.9 (5.3)	25.2 (6.7)	22.8 (3.0)	27.4 (5.2)	< 0.01
		Median	24	24	22	27	
		Min, Max	15, 44	15, 42	18, 32	17, 44	
Maternal education (years)	No formal education, illiterate	*n* (%)	170 (48)	6 (7)	58 (38)	106 (97)	< 0.01
	Literate, primary		107 (30)	49 (54)	57 (37)	1 (1)	
	Secondary, university		76 (22)	35 (39)	39 (25)	2 (2)	
Married	Yes	*n* (%)	349 (98)	86 (93)	154 (100)	109 (100)	< 0.01
	No		6 (2)	6 (7)	0 (0)	0 (0)	
Parity	1 to 2	*n* (%)	202 (55)	55 (61)	107 (68)	40 (33)	< 0.01
	3 to 4		98 (27)	16 (18)	48 (30)	34 (28)	
	5+		68 (18)	19 (21)	3 (2)	46 (38)	
Child food consumption at 36 months							
Grains, roots and tubers	Yes	*n* (%)	371 (100)	92 (100)	159 (100)	120 (100)	N/A
Fruits and vegetables	Yes	*n* (%)	341 (92)	92 (100)	141 (89)	108 (90)	< 0.01
	No		30 (8)	0 (0)	18 (11)	12 (10)	
Legumes and nuts	Yes	*n* (%)	155 (42)	84 (91)	65 (41)	6 (5)	< 0.01
	No		216 (58)	8 (9)	94 (59)	114 (95)	
Eggs	Yes	*n* (%)	101 (27)	18 (20)	28 (18)	55 (46)	< 0.01
	No		270 (73)	74 (80)	131 (82)	65 (54)	
Meat, fish or insects	Yes	*n* (%)	104 (28)	57 (62)	4 (3)	43 (36)	< 0.01
	No		267 (72)	35 (38)	155 (97)	77 (64)	
Milk and dairy	Yes	*n* (%)	284 (77)	21 (23)	143 (90)	120 (100)	< 0.01
	No		87 (23)	71 (77)	16 (10)	0 (0)	
Young Child Feeding Index components							
Modified Minimum Dietary	Yes	*n* (%)	216 (58)	66 (72)	72 (45)	78 (65)	< 0.01
Diversity	No		155 (42)	26 (28)	87 (55)	42 (35)	
Modified Minimum Meal	Yes	*n* (%)	297 (80)	55 (60)	147 (92)	95 (79)	< 0.01
Frequency	No		74 (20)	37 (40)	12 (8)	25 (21)	
Modified Minimum Acceptable	Yes	*n* (%)	174 (47)	43 (47)	66 (42)	65 (54)	0.11
Diet	No		197 (53)	49 (53)	93 (58)	55 (46)	
Child growth at 36 months							
Height Z-score		*N*	354	86	158	110	
		Mean (SD)	−2.36 (1.19)	−2.34 (1.60)	−2.31 (0.95)	−2.44 (1.12)	0.56
		Median	−2.28	−2.38	−2.24	−2.37	
		Min, Max	−5.69, 0.84	−5.69, 0.84	−5.19, −0.18	−5.31, 0.42	
Weight Z-score		*N*	363	91	159	113	
		Mean (SD)	−1.54 (1.13)	−0.62 (0.99)	−1.87 (1.01)	−1.81 (1.00)	< 0.01
		Median	−1.55	−0.81	−1.80	−1.69	
		Min, Max	−5.05, 2.33	−3.01, 2.33	−5.05, 0.61	−4.60, 0.61	
Weight-for-Height Z-score		*N*	354	86	158	110	
		Mean (SD)	−0.29 (1.50)	1.00 (1.53)	−0.78 (1.22)	− 0.58 (1.26)	< 0.01
		Median	−0.40	0.97	−0.93	−0.37	
		Min, Max	−4.56, 4.05	−3.02, 4.05	− 3.72, 2.70	− 4.56, 2.13	
Head Circumference Z-sore		*N*	359	91	156	112	
		Mean (SD)	−0.86 (1.39)	0.42 (1.11)	−1.64 (0.96)	−0.79 (1.31)	< 0.01
		Median	−1.03	0.35	−1.78	−0.74	
		Min, Max	−4.66, 3.18	−2.09, 3.18	− 4.63, 1.00	− 4.66, 1.79	
Stunting	Yes	*n* (%)	218 (59)	48 (52)	96 (60)	74 (62)	0.33
	No		153 (41)	44 (48)	63 (40)	46 (38)	
Wasting	Yes	*n* (%)	44 (12)	4 (4)	25 (16)	15 (13)	0.03
	No		327 (88)	88 (96)	134 (84)	105 (88)	

*P-values test difference between sites by chi-square, Fisher’s exact and Kruskal Wallis tests

All children in the cohort were said to be consuming grains, roots and tubers with the majority in each country also consuming fruits and vegetables (Table 1). Children from Zambia were more likely to have diets high in legumes or nuts (91%) and meat, fish or insects (62%) than children in India (41% and 3%, respectively) or Pakistan (5% and 36%, respectively) (*p* < 0.01). However, milk and other dairy were consumed less frequently by children in Zambia (23%) compared to children in India (90%) and Pakistan (100%) (*p* < 0.01). A significantly smaller proportion of children from India (45%) received the mMDD compared to Pakistan (65%) and Zambia (72%) (*p* < 0.01). On the other hand, a significantly larger proportion of children from India (92%) met the mMMF compared to Pakistan (79%) and Zambia (60%) (*p* < 0.01). About half (47%) of the cohort met the mMAD. Overall, children from Zambia tended to have higher WAZ-, WHZ-, and HCZ-scores (*p* < 0.01) and were less likely to experience wasting (*p* = 0.03) whereas children from India had the lowest HCZ-scores (*p* < 0.01) (Table 1).

MDI and PDI scores were measured in 369 of 371 (99%) children studied. Two children scored within normal limits for the 12- and 24-month evaluations but failed to complete the 36-month BSID-II evaluation. Among the 369 children evaluated, no significant associations were found between MDI and feeding practices or specific food groups. However, consumption of legumes or nuts was significantly associated with lower PDI scores in Pakistan. Although differences were not statistically significant, higher adjusted mean MDI and PDI scores were observed in those children consuming animal-source foods (eggs, dairy, and meat, fish or insects) (Table 2).

**Table 2:** Unadjusted and adjusted means for Bayley Scales of Infant Development measures at 36 months

			Mental Developmental Index (MDI)			Psychomotor Developmental Index (PDI)		
Measures		*N* ^a^	Mean ± SD (unadjusted)	Mean (adjusted)^b^	*P* value^b^	Mean ± SD (unadjusted)	Mean (adjusted)^b^	*P* value^b^
Exclusively breastfed first 6 months	Yes	230	94.25 ± 14.18	97.47	0.27	101.73 ± 15.07	101.80	0.61
	No	139	96.39 ± 13.96	95.43		100.40 ± 17.58	100.57	
Legumes and nuts	Yes	155	99.00 ± 12.05	95.62	0.35	105.02 ± 15.42	–	–
	No	214	92.20 ± 14.83	97.32		98.48 ± 15.97	–	
Zambia	Yes	84	–	–	–	105.08 ± 13.00	101.99	0.48
	No	8	–			101.88 ± 6.36	97.98	
India	Yes	65	–	–	–	106.60 ± 15.42	101.25	0.61
	No	92	–			95.63 ± 13.95	102.74	
Pakistan	Yes	6	–	–	–	87.00 ± 32.47	85.05	0.01
	No	114	–			100.54 ± 17.60	100.44	
Eggs	Yes	101	97.08 ± 14.92	96.98	0.62	102.87 ± 15.48	102.19	0.46
	No	268	94.29 ± 13.76	96.28		100.60 ± 16.25	100.85	
Meat, fish or insects	Yes	104	100.19 ± 12.21	98.04	0.13	104.39 ± 15.23	103.54	0.08
	No	265	93.04 ± 14.33	95.67		99.98 ± 16.22	100.03	
Milk and dairy	Yes	282	94.53 ± 14.45	96.74	0.57	100.97 ± 16.84	101.67	0.47
	No	87	96.77 ± 12.93	95.58		102.06 ± 13.23	99.74	
Modified Minimum Dietary Diversity	Yes	216	98.57 ± 13.10	96.87	0.38	103.97 ± 16.00	101.43	0.69
	No	153	90.09 ± 14.06	95.73		97.35 ± 15.36	100.76	
Modified Minimum Meal Frequency	Yes	295	94.79 ± 14.39	96.51	0.87	101.61 ± 15.86	101.57	0.44
	No	74	96.14 ± 13.03	96.27		99.70 ± 16.81	100.05	
Modified Minimum Acceptable Diet	Yes	174	98.41 ± 13.42	96.55	0.87	104.24 ± 15.93	101.21	0.98
	No	195	92.07 ± 14.09	96.36		98.54 ± 15.72	101.16	

^a^Numbers are among the 369 children with non-missing BSID-II scores. BSID-II scores were missing for 2/371 (0.5%) children [India: 2/159 (1.3%)]

^b^BSID-II means were estimated using linear models fit to each Bayley index score and adjusted for SES, site, intervention group, resuscitation status, evaluator, sex, exclusively breastfed first 6 months, birth weight, preterm status, maternal age, and maternal education level. Statistical significance for a difference in means for those with and without each food consumption measure was determined by the F test. Tests of whether the association between measure and outcome differed by site were conducted. Interactions involving milk and dairy could not be tested as all participants from Pakistan received milk and dairy on an average day. Interactions with site were significant for legume/nut consumption (*p* = 0.01); PDI score means are shown by site for this measure

Children who received the mMMF had significantly higher WHZ-scores, but not WAZ- or HAZ-scores, than those who did not (adjusted mean WHZ: 0.16 vs − 0.28, *p* = 0.02) (Table 3). Likewise, children who received the mMAD had significantly higher WHZ-scores, but not WAZ- or HAZ-scores (adjusted mean WHZ: 0.20 vs − 0.08, *p* = 0.05) (Table 3). HCZ-scores did not differ significantly between those who did and did not receive mMAD (Table 4). However, the association between flesh food consumption and HCZ-scores differed significantly by country [egg consumption and HCZ-score interaction (*p* = 0.01); meat/fish/insect consumption and HCZ-score interaction (p = 0.05)] (Table 4). Children from Pakistan who consumed eggs showed significantly higher HCZ-scores than those who did not (adjusted mean: − 0.31 vs − 1.24, *p* < 0.01), yet these same children were the only group with significantly lower HCZ-scores associated with meat, fish or insect consumption (adjusted mean: − 1.30 vs − 0.68, *p* < 0.01).

**Table 3:** Unadjusted and adjusted means for 36 month anthropometric measures

			Height Z-Score^a^			Weight Z-Score^a^			Weight-for-Height Z-Score^a^		
Measures		*N* ^b^	Mean ± SD (unadjusted)	Mean (adjusted)^c^	*P* value^c^	Mean ± SD (unadjusted)	Mean (adjusted)^c^	*P* value^c^	Mean ± SD (unadjusted)	Mean (adjusted)^c^	*P* value^c^
Exclusively breastfed first 6 months	Yes	223	−2.30 ± 1.22	− 2.30	0.19	−1.39 ± 1.22	−1.32	0.72	−0.15 ± 1.61	− 0.04	0.41
	No	131	− 2.47 ± 1.12	−2.56		−1.80 ± 0.90	−1.38		− 0.52 ± 1.26	0.15	
Legumes and nuts	Yes	148	−2.41 ± 1.35	−2.58	0.07	− 1.23 ± 1.27	− 1.41	0.44	0.20 ± 1.63	0.13	0.41
	No	206	−2.32 ± 1.06	−2.28		−1.77 ± 0.96	−1.30		−0.64 ± 1.29	−0.02	
Eggs	Yes	93	−2.46 ± 1.07	−2.46	0.78	−1.57 ± 1.03	−1.29	0.49	−0.24 ± 1.32	0.20	0.26
	No	261	−2.33 ± 1.23	−2.42		−1.52 ± 1.16	−1.38		−0.31 ± 1.56	0.01	
Meat, fish or insects	Yes	95	−2.39 ± 1.51	−2.42	0.92	−1.12 ± 1.19	−1.32	0.77	0.25 ± 1.81	0.04	0.92
	No	259	−2.35 ± 1.05	−2.44		−1.70 ± 1.07	−1.36		−0.48 ± 1.32	0.06	
Milk and dairy	Yes	270	−2.38 ± 1.11	−2.46	0.61	−1.73 ± 1.10	−1.34	0.81	−0.55 ± 1.37	0.09	0.66
	No	84	−2.28 ± 1.40	−2.34		−0.91 ± 0.99	−1.39		0.56 ± 1.59	−0.03	
Modified Minimum Dietary Diversity	Yes	203	−2.41 ± 1.25	−2.48	0.36	−1.44 ± 1.20	−1.33	0.66	−0.09 ± 1.53	0.15	0.12
	No	151	−2.30 ± 1.10	−2.36		−1.68 ± 1.01	−1.38		−0.55 ± 1.42	−0.09	
Modified Minimum Meal Frequency	Yes	284	−2.41 ± 1.15	−2.50	0.12	−1.59 ± 1.15	−1.32	0.35	− 0.31 ± 1.52	0.16	0.02
	No	70	−2.16 ± 1.32	−2.23		−1.31 ± 1.03	−1.45		− 0.21 ± 1.43	− 0.28	
Modified Minimum Acceptable Diet	Yes	164	−2.47 ± 1.22	−2.52	0.18	−1.51 ± 1.23	−1.33	0.65	− 0.12 ± 1.60	0.20	0.05
	No	190	−2.27 ± 1.15	−2.35		−1.56 ± 1.04	−1.38		−0.43 ± 1.40	−0.08	

^a^Z-scores deemed implausible according to WHO criteria were set to missing. This includes: WAZ < -6SD or > 5SD; HAZ < -6SD or > 6SD; WHZ < -5SD or > 5SD

^b^ N includes those with non-missing height and weight-for-height measurements (*N* = 354). There were additional weight measurements available (*N* = 363)

^c^Adjusted means were estimated using linear models fitting each anthropometric outcome and adjusted for SES, site, intervention group, resuscitation status, sex, exclusively breastfed first 6 months, birth weight, preterm status, maternal age, and maternal education level. Statistical significance for a difference in means for those with and without each food consumption measure was determined by the F test. Tests of whether the association between measure and outcome differed by site were conducted with none found to be significant. Interactions involving milk and dairy could not be tested as all participants from Pakistan received milk and dairy on an average day

**Table 4:** Unadjusted and adjusted means for head circumference Z-scores at 36 months

			Head Circumference Z-Score^a^		
Measures		*N* ^b^	Mean ± SD (unadjusted)	Mean (adjusted)^c^	*P* value^c^
Exclusively breastfed first 6 months	Yes	228	−0.82 ± 1.45	−0.81	0.92
	No	131	−0.92 ± 1.27	−0.79	
Legumes and nuts	Yes	153	−0.55 ± 1.51	−0.92	0.15
	No	206	− 1.08 ± 1.25	−0.69	
Eggs	Yes	95	−0.56 ± 1.28	–	–
	No	264	−0.96 ± 1.41		
Zambia	Yes	18	0.32 ± 1.29	0.20	0.94
	No	73	0.44 ± 1.07	0.18	
India	Yes	28	−1.62 ± 0.92	− 1.68	0.74
	No	128	−1.65 ± 0.97	−1.75	
Pakistan	Yes	49	−0.28 ± 1.05	−0.31	< 0.01
	No	63	−1.19 ± 1.35	− 1.24	
Meat, fish or insects	Yes	101	−0.33 ± 1.32	–	–
	No	258	−1.06 ± 1.36		
Zambia	Yes	57	0.27 ± 0.97	0.07	0.13
	No	34	0.66 ± 1.29	0.44	
India	Yes	4	−0.77 ± 1.24	− 0.91	0.12
	No	152	−1.67 ± 0.94	−1.78	
Pakistan	Yes	40	−1.14 ± 1.34	−1.30	< 0.01
	No	72	−0.60 ± 1.26	− 0.68	
Milk and dairy	Yes	273	−1.16 ± 1.28	−0.87	0.22
	No	86	0.11 ± 1.29	−0.61	
Modified Minimum Dietary Diversity	Yes	209	−0.74 ± 1.42	−0.86	0.27
	No	150	−1.01 ± 1.33	−0.72	
Modified Minimum Meal Frequency	Yes	287	−0.97 ± 1.32	−0.77	0.32
	No	72	−0.41 ± 1.54	−0.92	
Modified Minimum Acceptable Diet	Yes	168	−0.79 ± 1.36	−0.78	0.65
	No	191	−0.91 ± 1.41	−0.83	

^a^Z-scores deemed implausible (<-5SD or > 5SD) according to WHO criteria were set to missing

^b^ N includes those with non-missing head circumference measurements

^c^Adjusted means were estimated using linear models fitting head circumference Z score and adjusted for SES, site, intervention group, resuscitation status, sex, exclusively breastfed first 6 months, birth weight, preterm status, maternal age, and maternal education level. Statistical significance for a difference in means for those with and without each food consumption measure was determined by the F test. Tests of whether the association between each measure and the outcome differed by site were conducted. Interactions with site were significant for egg consumption (*p* = 0.01) and for meat, fish, or insects (*p* = 0.05); head circumference Z score means are shown by site for these measures

The prevalence of children with wasting was lower in those who received mMAD compared to those who did not (9% vs. 14%), but the difference was not statistically significant (Table 5). The only food group significantly associated with lower risk of wasting was egg consumption [adjusted RR (95% CI): 0.37 (0.15, 0.89), *p* = 0.03]. Yet, none of the YCF indicators nor any food groups were found to be associated with stunting.

**Table 5:** Unadjusted and adjusted relative risks of stunting and wasting for 36 month children

		Stunting^a^					Wasting^b^				
Characteristics/Measures			Unadjusted		Adjusted^c^			Unadjusted		Adjusted^c^	
		*n* (%)^d^	Relative Risk (95% CI)	*P* value^e^	Relative Risk (95% CI)	*P* value^e^	*n* (%)^d^	Relative Risk (95% CI)	*P* value^e^	Relative Risk (95% CI)	*P* value^e^
Sex	Male	134 (61)	1.07 (0.81,1.40)	0.63	1.14 (0.96,1.36)	0.12	31 (14)	1.60 (0.84,3.05)	0.16	1.61 (0.88,2.93)	0.12
	Female	84 (58)	REF		REF		13 (9)	REF		REF	
SES tercile	Low	110 (63)	1.72 (0.95,3.12)	0.07	1.42 (0.87,2.31)	0.16	25 (14)	1.56 (0.47,5.18)	0.47	1.17 (0.35,3.96)	0.80
	Medium	96 (59)	1.63 (0.89,2.97)	0.11	1.48 (0.92,2.39)	0.11	16 (10)	1.09 (0.32,3.73)	0.90	0.79 (0.23,2.68)	0.71
	High	12 (36)	REF		REF		3 (9)	REF		REF	
Legumes and nuts	Yes	89 (57)	0.96 (0.73,1.26)	0.78	1.10 (0.89,1.35)	0.37	14 (9)	0.65 (0.34,1.23)	0.18	0.95 (0.46,1.99)	0.90
	No	129 (60)	REF		REF		30 (14)	REF		REF	
Eggs	Yes	61 (60)	1.04 (0.77,1.40)	0.80	1.01 (0.84,1.22)	0.90	5 (5)	0.34 (0.14,0.87)	0.02	0.37 (0.15,0.89)	0.03
	No	157 (58)	REF		REF		39 (14)	REF		REF	
Meat, fish or insects	Yes	55 (53)	0.87 (0.64,1.18)	0.36	0.89 (0.71,1.13)	0.34	10 (10)	0.76 (0.37,1.53)	0.43	1.17 (0.55,2.48)	0.68
	No	163 (61)	REF		REF		34 (13)	REF		REF	
Milk and dairy	Yes	171 (60)	1.11 (0.81,1.54)	0.51	0.96 (0.70,1.31)	0.78	39 (14)	2.39 (0.94,6.06)	0.07	1.31 (0.56,3.06)	0.54
	No	47 (54)	REF		REF		5 (6)	REF		REF	
Modified Minimum Dietary Diversity	Yes	125 (58)	0.96 (0.74,1.26)	0.79	0.99 (0.84,1.17)	0.91	18 (8)	0.50 (0.27,0.91)	0.02	0.63 (0.35,1.15)	0.13
	No	93 (60)	REF		REF		26 (17)	REF		REF	
Modified Minimum Meal Frequency	Yes	179 (60)	1.14 (0.81,1.62)	0.45	1.12 (0.89,1.41)	0.32	36 (12)	1.12 (0.52,2.41)	0.77	0.88 (0.43,1.80)	0.73
	No	39 (53)	REF		REF		8 (11)	REF		REF	
Modified Minimum Acceptable Diet	Yes	103 (59)	1.01 (0.78,1.32)	0.92	1.01 (0.86,1.19)	0.89	16 (9)	0.65 (0.35,1.20)	0.16	0.74 (0.42,1.33)	0.32
	No	115 (58)	REF		REF		28 (14)	REF		REF	

^a^Stunting was defined as having −6 ≤ height Z-score < −2

^b^Wasting was defined as having −5 ≤ weight-for-height Z-score < −2

^c^Relative risks were estimated using Poisson regression models with robust variance estimators and adjusted for SES, site, intervention group, resuscitation status, sex, exclusively breastfed first 6 months, birth weight, preterm status, maternal age, and maternal education level

^d^Number and percent who were determined to have stunting or wasting, respectively

^e^Statistical significance was determined using the Wald chi-square test

## Discussion

In a cohort of 371 children aged 36 months of age from rural communities in India, Pakistan, and Zambia, we found 47% of the children met the mMAD. Mean WHZ-scores were significantly higher for children who received either the mMMF or mMAD compared to those who did not and notably, egg consumption was associated with a significant decreased risk of wasting. However, adjusted mean HAZ-, WAZ- and HCZ-scores were not statistically different between those who did and did not receive mMAD. Likewise, there was no significant association between these dietary practices and MDI and PDI.

While the majority of children (80%) met the mMMF, inclusion of either legumes/nuts, dairy, eggs, or meat/fish/insects was required to meet the mMDD, and thus the mMAD. As expected, due to the majority of the participants in India being lacto-vegetarians, very few children in India consumed animal-flesh foods (3%) and only a minority consumed eggs (18%) and legumes or nuts (41%) resulting in a smaller percentage reaching the mMAD. Indian children also had the lowest WHZ-scores and the highest prevalence of wasting. While encouraging a diet higher in animal-flesh foods or eggs may not be culturally appropriate for this group, increasing consumption of legumes and/or nuts could help them meet the mMDD and, therefore, mMAD. In contrast, Zambian children were more likely to be consuming meat, fish or insects (62%) and legumes or nuts (91%), had the highest WHZ-scores and the lowest prevalence of wasting. While causal inferences cannot be made, the significant positive overall associations reported here between WHZ-scores and mMAD highlight the importance of providing optimal feeding practices for young children, including achieving the required number of meals as well as a variety of foods in the diet.

Of interest was the association between egg consumption and decreased risk of wasting found here. Eggs are rich in amino acids, especially leucine which stimulates muscle protein synthesis and may potentially explain this protective effect. In addition, consumption of eggs and other animal source foods was found in children with higher neurodevelopment scores, although not statistically significant. These foods provide a concentrated source of dietary macro- and micronutrients essential for optimal growth, immune response and cognitive function, including protein, iron, zinc, and vitamin B12 [9, 19, 20]. Iron and zinc are critical micronutrients for appropriate development of the hippocampus and prefrontal cortex in the first 1000 days of life [21, 22] and vitamin B12, a key factor for normal brain and nervous system function, is only found in animal-source foods.

However, mixed findings were observed between micronutrient-rich food groups and HCZ-scores, specifically in Pakistan. In these children, better HCZ-scores were significantly associated with egg consumption, in contrast to a negative association with meat, fish, or insect consumption. While these results were unexpected, differences in demography and feeding habits may have contributed to these conflicting results, as well as potential inconsistencies in head circumference measurement.

The relationship between diet and child growth is complex and the lack of specificity and sensitivity of this qualitative dietary tool may also account for the variability found here, as well as the inability to identify other meaningful associations, which is in line with findings from a systematic review conducted by Jones and colleagues [23]. For example, a study from Senegal showed that HAZ was positively associated with the WHO indicators up to one year of age but less strongly as children became older, which may explain why we saw no significant results at 36 months of age between diet and linear growth [24]. Further, our results are in line with another study from Ghana which showed that these indicators better explain WHZ-scores than HAZ-scores for young children because the tool reflects current diet rather than habitual intake [25].

There was a significant association between legume/nut consumption and lower PDI scores in Pakistan (*p* = 0.01). However, given that only six children at this site were reportedly consuming legumes or nuts on a typical day, these findings may not be reliable. It is possible we found no other significant associations between diet and MDI and PDI scores because of the difficulty of assessing neurodevelopment in young children. Yet, to our knowledge, this is the first study to explore the relationship between YCF indicators and BSID-II MDI and PDI scores at 36 months and a longitudinal examination from an earlier age using a quantitative dietary approach may provide more meaningful results.

We acknowledge the WHO YCF indicators were designed for younger children, but suggest our modifications were age-appropriate and consistent with guidelines for MMF and MDD. Long term growth is influenced by feeding practices beginning from birth. A limitation of this study is that data on dietary practices were only collected at the final 36-month visit. As a result, this cross-sectional design, prohibited us from assessing the impact of earlier feeding practices, such as the introduction of complementary foods, on growth and development. However, data on nutrition may not change over time as the food types in these settings are typically limited. Lastly, another limitation of our study was that different sites used different measurement techniques (i.e. type of scale utilized, and standing height versus recumbent length) and measurement bias and inter-observer bias can not be ruled out.

## Conclusion

This study demonstrated positive associations between meeting either the mMMF or mMAD and favorable WHZ-scores, and more specifically a decreased risk of wasting with egg consumption. For cohorts such as our Indian site who are primarily vegans and vegetarians, increased consumption of legumes and nuts may help these populations meet the mMDD and, thus the mMAD. While the WHO YCF indicators may be valuable tools on a population level, additional specific longitudinal measures of dietary intake are needed to fully assess feeding practices and their relationship with linear growth and neurodevelopment in children.

## Abbreviations

BRAIN-HIT: Brain Research to Ameliorate Impaired Neurodevelopment Home-based Intervention Trial WHO World Health Organization BSID-II Bayley Scores of Infant Development – Second Edition MDI Mental Development Index PDI Psychomotor Development Index EDI Early developmental intervention mMDD modified Minimum Dietary Diversity mMMF modified Minimum Meal Frequency mMAD modified Minimum Acceptable Diet LMIC low- and low-middle-income countries
